# Supplementary material for: Preparation of iron(IV) nitridoferrate Ca4FeN4 through azide-mediated oxidation under high-pressure conditions
Source: Nat Commun. 2021 Jan 25;12:571. doi: 10.1038/s41467-020-20881-y (PMC7835361; doi:10.1038/s41467-020-20881-y)
Supplement: Supplementary file 1 — Supplementary Information [file 41467_2020_20881_MOESM1_ESM.pdf]

Supplementary Information

Title: Preparation of iron(IV) nitridoferrate Ca_4FeN_4 through azide-mediated oxidation under high-pressure conditions

Authors: Simon D. Kloß[1*], Arthur Haffner[2], Pascal Manuel[3], Masato Goto[4], Yuichi Shimakawa[4], J. Paul Attfield[1*]

Affiliation

1 University of Edinburgh, Centre for Science at Extreme Conditions and School of Chemistry, Edinburgh EH9 3FD, UK

2 Ludwig-Maximilians-University Munich, Department Chemistry, 81377 Munich, Germany

3 ISIS Neutron Source, STFC Rutherford Appleton Laboratory, Didcot, Oxfordshire, OX11 0QX, UK

4 Institute for Chemical Research, Kyoto University, Uji, Kyoto 611-0011, Japan

Table of Contents

1. Supplementary Methods 3

  1.1 Synthesis 3

  1.2 Diffraction 4

  1.3 Magnetometry 5

  1.4 ^{57}Fe-Mössbauer 5

  1.5 Scanning Electron Microscopy 5

2. Supplementary Discussion 6

  2.1 Single-crystal diffraction 6

  2.2 Detailed structure description 6

  2.3 Additional discussion of the bonding situation 7

  2.4 Details on neutron powder diffraction 8

  2.5 Impurity effects 9

3. Supplementary Figures 11

4. Supplementary Tables 17

5. Supplementary References 23

1. Supplementary Methods

1.1 Synthesis

Ca_4FeN_4. The title compound was prepared following the stoichiometry of equation (1) from calcium nitride Ca_3N_2 (99.9 %, AlfaAesar), iron nitride Fe_2N, and sodium azide NaN_3 (99.99%, Sigma-Aldrich). Reactions were carried out between 4 and 8 GPa and between 800 and 1200 °C, and optimum synthesis conditions were found to be 6 GPa and ca. 1200 °C with 60/300/120 minutes of ramp up, dwell, and ramp down, respectively. Under the inert conditions of an Ar-filled glovebox ($c(O_2/H_2O) < 1$ ppm), all compounds were ground and transferred into a copper capsule (0.025 mm thickness, 99.999 %, Puratronic®, AlfaAesar), which was then placed in a h-BN crucible that was subsequently placed inside the sample octahedron. The reaction conditions were achieved by a Voggenreither 1000 t large volume press (Mainleus, Germany) with multianvil technique employing an 18/11 octahedron-within-cubes payload. The pressure medium consisted of a Cr_2O_3-doped MgO-octahedron (Ceramic Subtrates & Components, Isle of Wight, U.K) with 18mm edge-length. Heating was enabled by resistance heating using two graphite sleeves (Schunk, Heuchelheim, Germany) to minimize the temperature gradient. Additional information about the setup can be found in literature.[1]

Caution: NaN_3 is highly toxic and can undergo rapid decomposition when exposed to high temperatures.

Approximately 50 mg samples were obtained from each experiment, and these were assessed by powder X-ray diffractogram as shown in Supplementary Fig. 3. One sample was used for single crystal diffraction, EDX, and magnetisation measurements, another sample was used for neutron and powder X-ray diffraction, and several samples were combined for ^{57}Fe-Mössbauer measurements. This and possible part-decomposition due to moisture sensitivity, accounts for the varying impurity contribution seen in different measurements, as discussed later.

Fe_2N. The starting material Fe_2N has been prepared by reaction of Fe metal powder with a constant flow of dried ammonia (5.0, Air Liquide) at 500 °C. The temperature ramps were 5 °C/min and dwell 20 h. The ammonolysis was repeated twice with intermittent

grinding of the obtained grey-metallic powder until the X-ray diffraction pattern showed only single-phase Fe_2N.

1.2 Diffraction

Single-crystal X-ray diffraction data of Ca_4FeN_4 were obtained with a Bruker D8 Venture diffractometer on single crystals mounted in glass capillaries (Hilgenberg, Malsfeld, Germany) under dried paraffin oil to prevent hydrolysis. Data collection, indexing, data reduction, and absorption correction were carried out with the APEX3 software.[2] Analysis of systematic absent reflections and space group determination was supported by the XPREP software.[3] Structure solution and refinement was carried out with the SHELX software implemented in WINGX.[4,5] Structures were visualized with VESTA.[6] Further information regarding the crystal structure can be obtained through the joint CCDC/FIZ Karlsruhe inorganic crystal structure database by quoting number CSD 2015297.

Powder diffraction was carried out with a STOE StadiP diffractometer equipped with a DECTRIS MYTHEN 1K Si-strip detector in modified Debye-Scherrer geometry. Samples were sealed in glass capillaries with an inner diameter of 0.5 mm (Hilgenberg, Malsfeld, Germany). Owing to the Fe-content of the sample, a Mo X-ray source was used with a Ge(111) monochromator singling out the Mo-$K_{\alpha1}$ radiation. Data were collected in the range from $2 < 2\theta < 71°$ and Rietveld refinement was carried out with the Topas Academic V4.1 software.[7] The background was handled with a shifted-Chebychev function, peak profiles with a fundamental parameter approach, and preferred orientation with spherical harmonics. Positions of the heavier atoms Fe and Ca were refined as well as the displacement parameters.

Neutron powder diffraction was carried out at the WISH beamline of the ISIS Neutron and Muon Source at temperatures of 1.5 and 50 K.[8] For the refinement, data collected between 12.1 and 1.4 Å on four detector banks were used. Rietveld refinement and magnetic structure solution and refinement were performed with GSAS-II.[9] The background was handled with shifted-Chebychev functions fitted to manually set points owing to strongly curved background and peak overlap. The background was subtracted before visualization of the fits in the figures. The magnetic structure was displayed with VESTA.[6]

1.3 Magnetometry

Magnetization curves $M(H,T)$ were recorded with a Quantum Design Physical Properties Measurement System (PPMS). Powdered samples of Ca_4FeN_4 were packed into polyethylene capsules and sealed with glue to prevent decomposition of the sample owing to moisture. Isothermal magnetization curves in the range of ±50 kOe were recorded at temperatures of 300, 40, 19, and 2 K, while the temperature dependent susceptibility was obtained at 30 kOe in the range of 2 to 300 K. The obtained magnetizations were corrected for the diamagnetic contribution of the capsule.

1.4 ^{57}Fe-Mössbauer

^{57}Fe-Mössbauer measurements were performed in transmission geometry with a constant-acceleration spectrometer using a ^{57}Co/Rh radiation source. The velocity scale and the isomer shift were determined with the relative values of α-Fe at room temperature. The spectra were fitted with Lorentzian functions by using the standard least-squares method. A small unidentified impurity was detected in the ^{57}Fe-Mössbauer spectra, which probably is related to the one detected by PXRD and magnetic measurements.

1.5 Scanning electron microscopy

A Zeiss EVO-Ma 10 scanning electron microscope was used for obtaining micrographs and electron energy dispersive (EDX) measurements. Selected crystallizes of the sample were placed on conductive and adhesive carbon tape and quickly inserted into the electron microscope, which was equipped with a field emission gun run at 15 keV and a Bruker X-Flash 410-M detector. Data were analysed with the QUANTAX 200 software package. Oxygen content was not taken into account owing to the short exposure in air and consequential hydrolysis. Samples were not sputtered owing to the hydrolysis.

2. Supplementary Discussion

2.1 Single-crystal diffraction

The dataset of Ca_4FeN_4 was indexed in an *I*-centred orthorhombic unit cell with pseudo-tetragonal metric a = 6.903(2), b = 6.919(3), and c = 22.552(8) Å and was solved with direct methods in space group *Ibca* (no. 73), which was unambiguously determined through examination of systematic absences, resulting in the heavy atom positions. Nitrogen positions were determined with difference Fourier maps. All atom positions were refined anisotropically and higher crystal symmetry was ruled out based on Platon suggestions and absent 4-fold rotation and rotoinversion symmetry elements.[10]

2.2 Detailed structure description

For a better understanding of the intricate structure, it can be formally subdivided into three different layers, each of them composed of one kind of CaN_6 octahedron stacked along c as shown in Figure 1a.

$Ca1N_6$ octahedra form the middle layer with equatorial N-atoms in one plane at height c = 0.5 forming an almost regular square tiling. The octahedra are interconnected via all-side edge-sharing in this equatorial plane. The axial N-atoms of the $Ca1N_6$ octahedra are also part of the $[FeN_3]^{5-}$ polygon and are dislocated from the ideal position of a regular octahedron through the structure directing influence of the strong and directed covalent Fe–N bonds. This structure directing and the more ionic Ca–N, which can tolerate larger bond angle distortions, are a characteristic motif of the Ca_4FeN_4 structure.

The $Ca2N_6$ octahedra of the second layer are connected via one common edge to the $Ca1N_6$ octahedra and via common corners to each other and the $[FeN_3]^{5-}$ units. The third layer is formed from $Ca3N_6$ octahedra that are connected via common faces and edges to the $Ca2N_6$ octahedra and each other, and via common edges to the $[FeN_3]^{5-}$ units. The $Ca3N_6$ octahedra show the largest bond angle variance (Supplementary Fig. 2, Supplementary Table 6) with respect to a regular octahedron and thus are a contrast to the more ordered region of the first and second layer, which is also mirrored by relatively large displacement ellipsoids of Ca3.[12]

Sandwiching layer 1 by layers 2 and 3 yields the smallest repeat unit of the structure (marked in Figure 1b). The automorphism group of this repeat unit contains an inversion centre by which layers 2 and 3 on top and bottom are related to each other. The repeat units themselves are stacked along *c* by inversion, or by the *a*-glide reflections perpendicular to the *c*-axis.

2.3 Additional discussion of the bonding situation

The $[FeN_3]^{5-}$ complex anion (Figure 1c) resides on a special position with a twofold rotation axis running through atomic sites Fe1 and N3 resulting in point symmetry C_{2v} for the trigonal-planar anion. The spin state of the Fe^{IV} ion and the relevant metal-ligand bonding can be rationalized along the general theoretical framework established for bonding in trigonal-planar coordination with monoatomic double-faced π-donors and the already existing examination of $[M^{III}N_3]^{n-}$ (M^{III} = V to Fe) anions.[13,14,15,16,17,18,19] The qualitative d-orbital splitting is shown in Figure 1d. While weak π-donors like oxides and halides usually lead to high-spin configuration, it was shown by ab initio calculations on $[M^{III}N_3]^{6-}$ complex anions with M^{III} = V, Cr, Fe that the strong π-bonding character of N leads to high energies of the π-antibonding e'' and e' orbitals, thus enabling low-spin configuration.[17–19] Owing to an even lower electron repulsion and higher crystal field splitting in Fe^{IV} than in Fe^{III}, a low-spin state is also assumed for $[FeN_3]^{5-}$ complex anions leading to occupied a'_1 and half-occupied e' orbitals and spin of $S = 1$ (Figure 1d).

Ca_4FeN_4 exhibits shorter Fe–N bond lengths than the known Ca nitridoferrate(III) $Ca_6Fe^{III}N_5$ with d_{Fe-N} = 1.769(15) Å but similar bond lengths are observed in nitridoferrates(III) of the higher homologues, Sr_3FeN_3 and Ba_3FeN_3 with d_{Fe-N} = 1.73(1) Å.[16,20] The shortening of the bond with respect to the $Ca_6Fe^{III}N_5$ is probably caused by a smaller ionic radius of Fe^{IV} and the removal of an electron from the antibonding e'' orbitals. The similar bond lengths observed in the A_3FeN_3 (A = Sr, Ba) compounds are probably owed to the lower electronegativity of Sr and Ba, which leads to a larger electron inductive effect and stronger Fe–N bond. Similar trends in bond length have been observed for the A_3CrN_3 (A = Ca, Sr, Ba) system.[14,21] The multiple Fe–N bonding in Ca_4FeN_4 is apparent when compared to amido-ligands (e.g. $Fe[N(SiMe_3)_2]_3$ d_{Fe-N} = 1.917(4) Å), which are single-faced π-donating, and bridging nitrido-ligands as in Fe_2N (d_{Fe-N} = 1.94 Å) that features vertex-sharing $[FeN_{3/6}]$ units.[22,23] The observed bond lengths thus mirror

the calculated bond order of 2 in this 16-electron $[FeN_3]^{5-}$ complex anion with three σ- and four π-bonds with two electrons in antibonding orbitals.

In 3d metal nitridometallates Ca is usually coordinated with CN = 5, though some examples for octahedral Ca-coordination have been reported in $Ca_{12}Mn_{19}N_{23}$ and $Ca_{133}Mn_{216}N_{260}$, which were prepared by gas-solid reaction at ambient pressure.[24]

2.4 Details on neutron powder diffraction

Owing to a maximum resolution of ca 1.1 Å obtained with the four detector banks at 50 K, the atom positions and displacement parameters obtained from refinement might not be reliable. Moreover, preferred orientation and bad overall crystallinity of the small plate-like crystallites complicated the refinement. Hence, the low temperature nuclear structure models were compared with the room temperature single-crystal structure with the COMPSTRU tool of the Bilbao crystallographic server, which indicated only slight structural changes (Supplementary Table 8). The largest changes are in the lateral x- and y-positions of Ca3 and N1, which also show the large displacement ellipsoids in the RT single-crystal model. This might indicate crystal stacking faults as can also be deduced from the plate-like morphology of the crystals. As the nuclear models, however, reasonably fit the data and do not show large distortions with respect to the single-crystal model, they were taken as a starting point for the elucidation of the magnetic structure.

For the determination of the magnetic cell, the difference of 1.5 and 50 K data was used because of overlap from an impurity with the (1 0 0) and (0 1 0) reflection position. That the impurity reflection does not belong to the structure model at high temperature was corroborated through single-crystal diffraction, which unambiguously shows reflection conditions *hkl* = 2*n* (*I*-centring), *hk*0 = 2*n* (−−*a*-glide), and 0*kl* = 2*n* (*b*−−-glide) leading to systematic absence of the (1 0 0) and (0 1 0) reflections. The magnetic cell was indexed with the same unit cell vectors as the nuclear model. The magnetic cell enlargement for the antiferromagnetic ordering is realized through the *klassengleiche* transformation of index *k*2 from *Ibca* to *Pbca* with loss of centring translations.

The determination of Bravais-class and subsequent Shubnikov-group was complicated by the pseudo-tetragonal metric of the unit cell, leading to overlapping reflections as indicated in Figure 4a. The (1 0 0) reflection gave the worse fits as its theoretical position

did not perfectly coincide with the observed reflection. A magnetic ordering parallel to the (1 0 0) planes was thus ruled out in favour of the better fitting (0 1 0) reflection leading to magnetic ordering parallel to the *bc*-plane. Such an ordering could be realized in several Shubnikov-groups but the fit in group *Pbc'a* gave the best fit. *Pbc'a* is non-standard setting of *Pb'ca* and was chosen to retain the nuclear structure setting.

2.5 Impurity effects

Impurity contributions are seen in the powder diffraction, magnetic, and Mössbauer data, and it is important to estimate their influence in particular on the reported magnetic properties.

Powder X-ray and neutron diffraction data contain unidentified impurity peaks up to 13% (Fig. S3) and 12 % (Fig. S4) of the maximum Ca_4FeN_4 peak integrated intensity, which serve as crude estimates of impurity phase proportion. Impurity effects in the magnetization data (Figs. 2, S6 and S7) are, however, rather small. A linear Curie-Weiss fit (Fig. 2) and constant Curie-Weiss paramagnetic moment (Fig. S7) close to the expected value for $S = 1$ Fe^{4+} are observed over a wide temperature range (80-300 K) showing that no large amounts of paramagnetic impurity are present. An observed low temperature paramagnetic tail is consistent with no more than 3% of a $S = ½$ impurity (Fig. S7), and M-H loops show that only a trace of ferromagnetic impurity is present, equivalent to ~0.01% Fe metal (Fig. S6). Taken together, these results indicate that the secondary phases observed in the powder diffraction data are mainly non-magnetic, in keeping with the 4:1 Ca:Fe ratio of metals and also Na in the bulk sample.

The Mössbauer sample is inconsistent with the magnetisation results as 14 atom-% of an Fe impurity is observed (Fig. 3), and any Fe-based phase would be likely to be para- or ferro- magnetic. This may be due to mixing of products of varying purity to make the Mössbauer sample and perhaps also partial decomposition of the highly air- and moisture- sensitive Ca_4FeN_4 during sample transfers or transport from Europe to Japan for this measurement. Inclusion of a 14 atom-% impurity doublet in the fits to the low temperature Mössbauer data enabled good fits of the magnetic sextet from Ca_4FeN_4 to be obtained.

The major magnetic features in the susceptibility, magnetic neutron and Mössbauer data may thus be assigned to Ca_4FeN_4 with high confidence, despite the presence of unidentified secondary phases.

3. Supplementary Figures

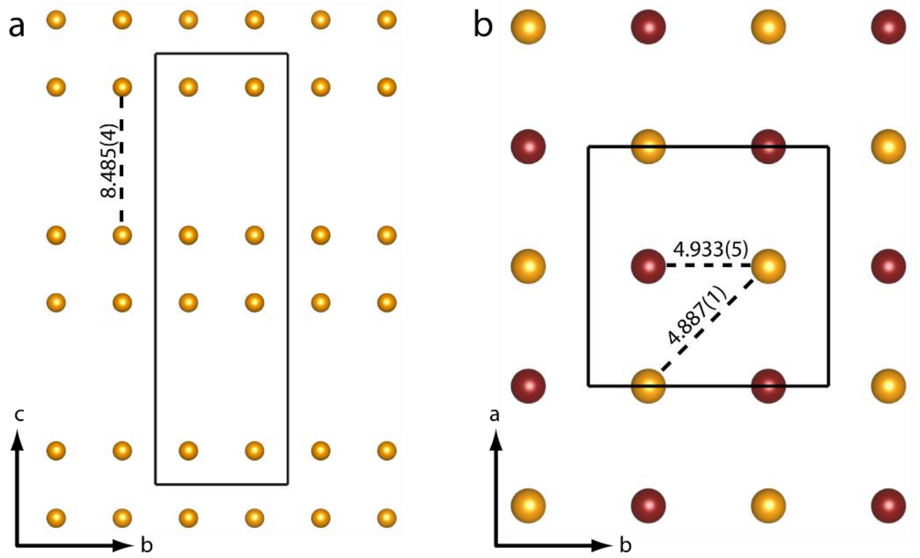

Supplementary Fig. 1. Fe sublattice in Ca_4FeN_4. **a** View parallel to the planes and **b** view perpendicular to the planes with Fe atoms at different *z*-positions differentiated through colouring. Fe–Fe distances are displayed in Å.

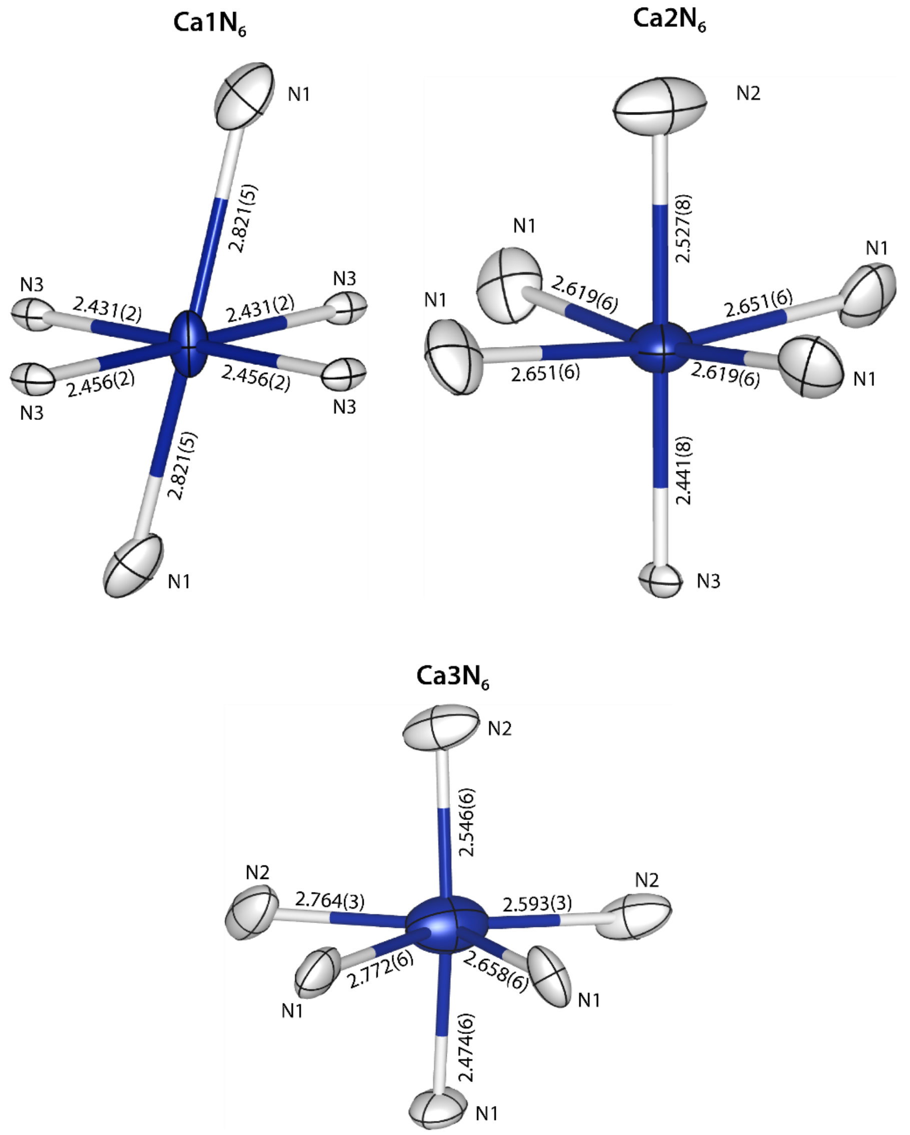

Supplementary Fig. 2. Ca coordination occurring in Ca_4FeN_4. Bond lengths are displayed in Å. Bond-angles are omitted for clarity and are displayed in Supplementary Table 5.

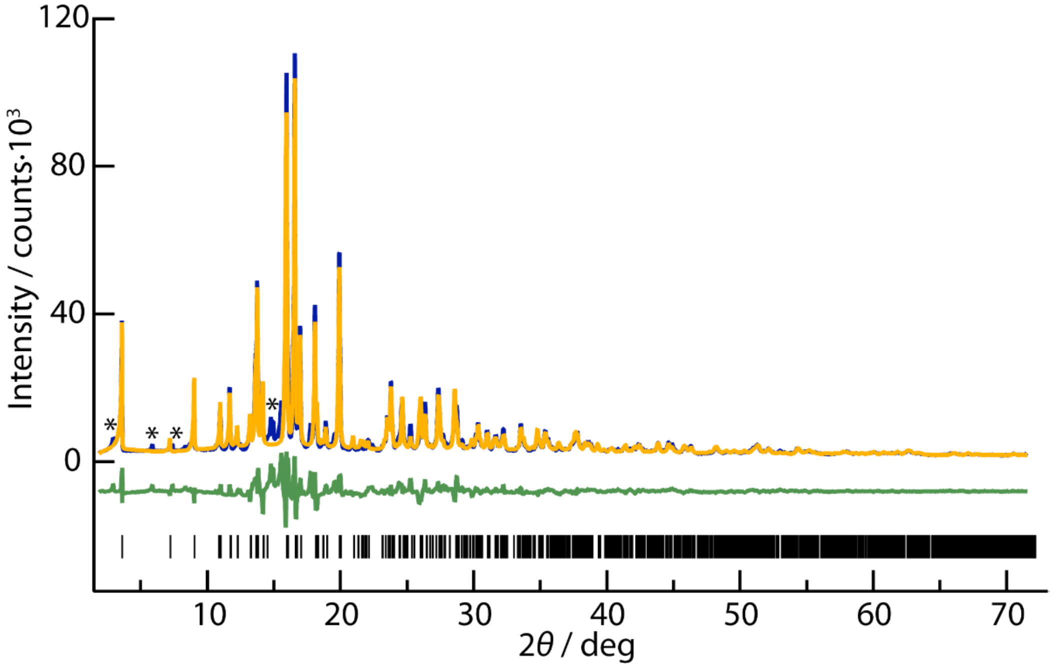

Supplementary Fig. 3. Rietveld refinement of laboratory X-ray powder diffraction data of a Ca_4FeN_4 sample. Collected data in blue, calculated model in orange, difference in green. Black ticks mark positions of theoretical Bragg reflections. Black asterisks mark the positions of the strongest impurity reflections (maximum impurity peak integrated intensity = 13% of maximum Ca_4FeN_4 peak).

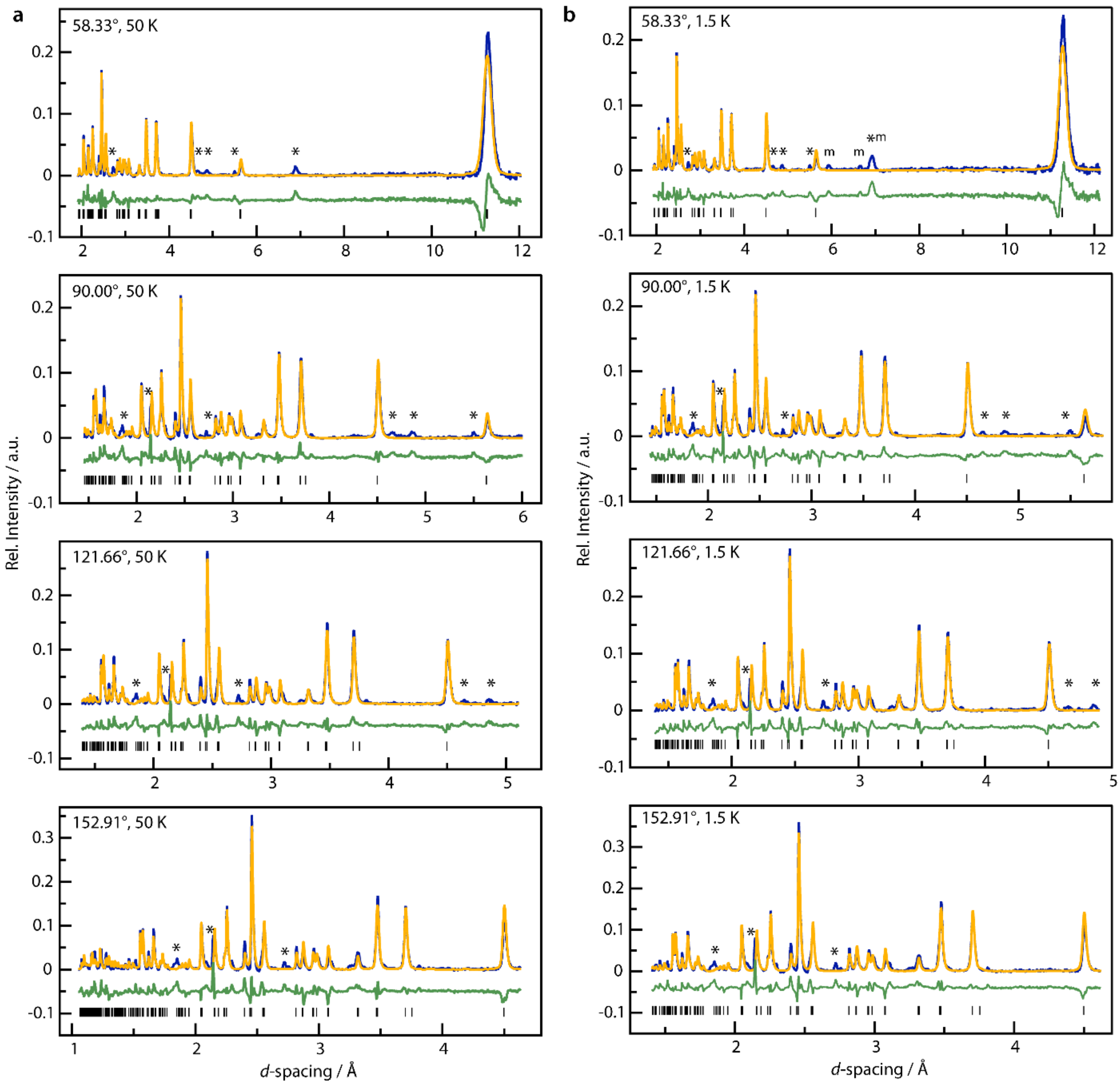

Supplementary Fig. 4. Rietveld refinement of neutron powder diffraction data of Ca_4FeN_4 obtained at the WISH beamline of the ISIS Neutron and Muon Source. Collected data in blue, calculated model in orange, difference in green. Black ticks mark positions of theoretical Bragg reflections. **a** data obtained at 50 K and **b** data at 1.5 K, the detectors are distinguished through their corresponding angle. Strongest impurity reflections marked by asterisks and magnetic reflections marked by **m** in the 1.5 K data (maximum impurity peak integrated intensity = 12 % of maximum Ca_4FeN_4 peak).

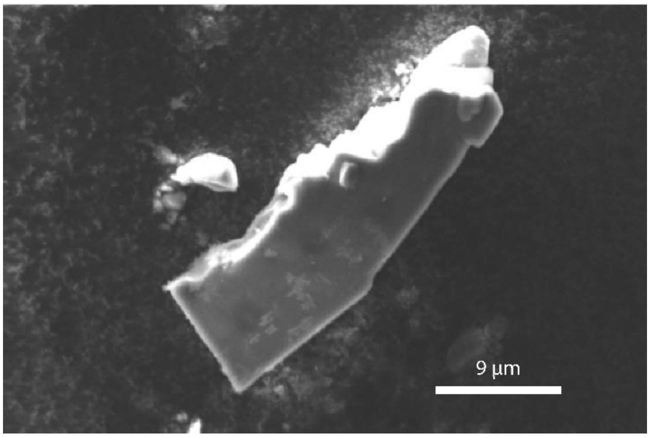

Supplementary Fig. 5: SEM micrograph of a Ca_4FeN_4 crystallite. The plate-like morphology and straight crystal edges are visible.

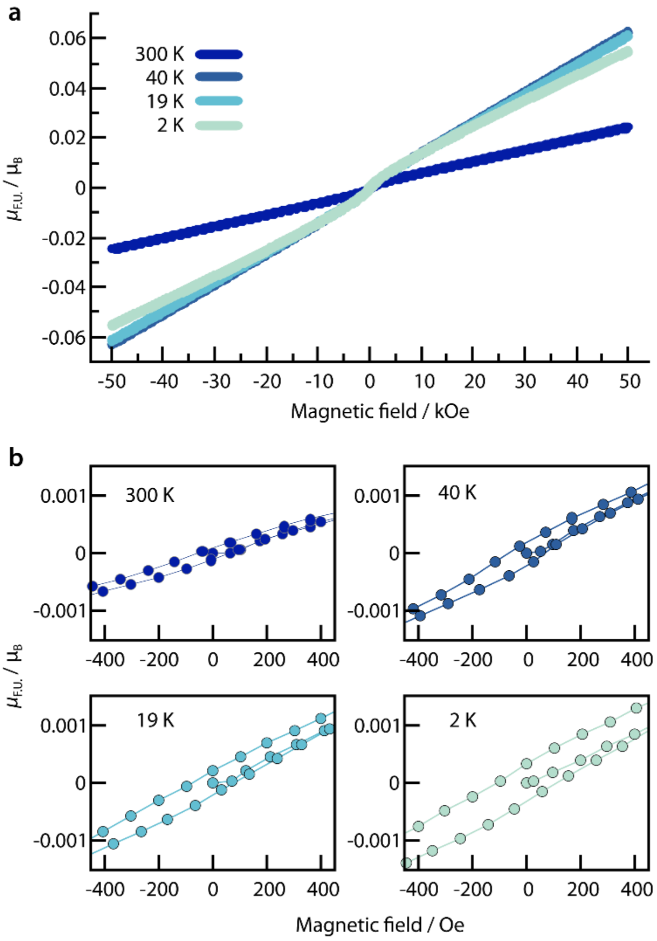

Supplementary Fig. 6: Isothermal magnetization of Ca_4FeN_4 obtained at 2, 19, 40, and 300 K in a field ranging from −50 to 50 kOe. **(a)** Magnetization $\mu_{F.U.}$ per Ca_4FeN_4 formula unit shown up to ±50 kOe. The magnetization curves obtained at 2 and 19 K lie below the 40 K measurement and slightly deviate from linear behaviour with increasing field indicating an

antiferromagnetic ordering in line with the temperature dependent susceptibility data. **(b)** Enlarged region of the four plots at low field. The small remanent magnetization (0.0003 μ_B at 2 K) and hysteresis show that only a trace of ferromagnetic impurity is present. This is most likely Fe metal, present at ~0.01% level.

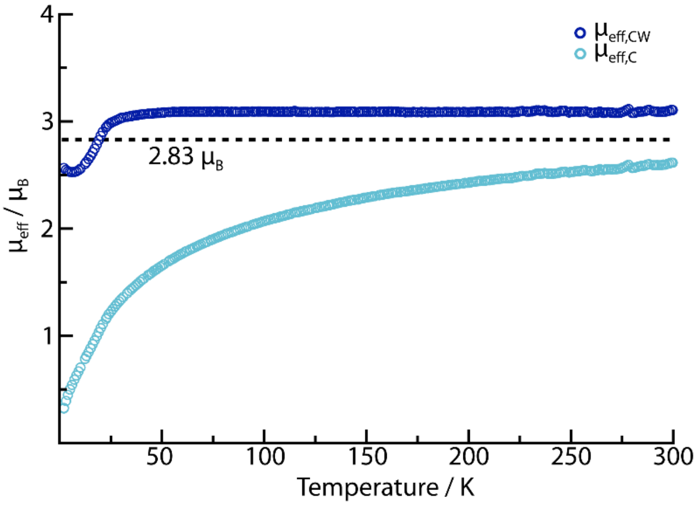

Supplementary Fig. 7: Effective magnetic moment vs. temperature. Dark blue points shows μ_{eff} obtained from the Curie-Weiss fit in the main text with $\mu_{eff,CW} = \sqrt{\chi_{mol} \cdot (T - \theta)/0.1246}$ where $\theta = -123$ K, and light blue points show μ_{eff} obtained through the Curie law with $\mu_{eff,C} = \sqrt{\chi_{mol} \cdot T/0.1246}$. Dashed line indicates 2.83 μ_B, the ideal spin-only μ_{eff} value for $S = 1$ systems. A small low temperature Curie tail is seen in the susceptibility data (Fig. 2), and the $\mu_{eff,C}$ variation here at lowest temperature shows that the Curie impurity contribution is no more than μ_{eff} ~0.3 μ_B, equivalent to 3% of a $S = ½$ impurity.

4. Supplementary Tables

Supplementary Table 1: Crystallographic data for Ca_4FeN_4.

**Crystal Data**	
Formula	Ca_4FeN_4
Formula mass, g·mol^{-1}	272.19
Crystal system	Orthorhombic
Space group	*Ibca* (no. 73)
Cell parameters, Å	a = 6.903(2)<br>b = 6.919(3)<br>c = 22.552(8)
Cell volume, Å^3	1077.2(7)
Formula units, Z	8
F(000)	1072
Calculated density ρ, g·cm^{-3}	3.357
Absorption coefficient μ, mm^{-1}	6.482
**Data Collection**	
Diffractometer	D8 Venture
Radiation	Mo-Kα
Temperature, K	273(2)
θ range, deg.	3.61 < θ <27.85
Total no. of reflections	5956
Independent reflections	622
Absorption correction	Semiempirical[11]
R_{int}, R_{sigma}	0.088, 0.053
**Refinement**	
Refined paramters	44
GOF	1.077
R_1 (all data), R_1 [$F^2 > 2\sigma(F^2)$]	0.0729, 0.0463
wR_2 (all data), wR_2 [$F^2 > 2\sigma(F^2)$]	0.0914, 0.0834
$\Delta\rho_{max}$, $\Delta\rho_{min}$, e·Å^3	0.98, −1.06

Supplementary Table 2: Atom positions of Ca_4FeN_4.

Atom	Wyckoff positions	Site symmetry	*x*	*y*	*z*	U_{eq} / Å^2	s.o.f.
Fe1	8*e*	..2	0	1/4	0.07815(5)	0.0076(3)	1
Ca1	8*d*	.2.	0.25	0.0025(3)	0	0.0095(4)	1
Ca2	8*e*	..2	0	1/4	0.39196(7)	0.0131(4)	1
Ca3	16*f*	1	0.3305(2)	0.0993(2)	0.30905(6)	0.0249(4)	1
N1	16*f*	1	0.1532(8)	0.0999(8)	0.1178(2)	0.0149(11)	1
N2	8*e*	..2	0	1/4	0.2799(3)	0.0189(17)	1
N3	8*e*	..2	0	1/4	0.0002(3)	0.0053(12)	1

Supplementary Table 3: Anisotropic displacement parameters for Ca_4FeN_4.

Atom	U_{11} / Å^2	U_{22} / Å^2	U_{33} / Å^2	U_{23} / Å^2	U_{13} / Å^2	U_{12} / Å^2
Fe1	0.0084(6)	0.0065(5)	0.0080(6)	0	0	0.0013(6)
Ca1	0.0057(7)	0.0051(7)	0.0178(9)	0	0.0001(8)	0
Ca2	0.0167(8)	0.0135(8)	0.0089(8)	0	0	−0.0004(10)
Ca3	0.0328(8)	0.0219(7)	0.0201(7)	−0.0027(6)	0.0050(7)	−0.0085(7)
N1	0.015(3)	0.013(3)	0.017(3)	0.003(2)	−0.007(2)	0.003(3)
N2	0.012(4)	0.034(5)	0.011(3)	0	0	−0.008(4)
N3	0.008(3)	0.005(3)	0.004(3)	0	0	−0.003(4)

Supplementary Table 4: Selected interatomic distances (in Å) occurring in Ca_4FeN_4.

Fe1–N1	1.731(5)	Ca2–N3	2.440(6)
Fe1–N1	1.731(5)	Ca2–N2	2.527(7)
Fe1–N3	1.759(6)	Ca2–N1	2.618(6)
Ca1–N3	2.4312(15)	Ca2–N1	2.618(6)
Ca1–N3	2.4312(15)	Ca2–N1	2.651(5)
Ca1–N3	2.4558(15)	Ca2–N1	2.651(6)
Ca1–N3	2.4558(15)	Ca3–N1	2.474(5)
Ca1–N1	2.820(6)	Ca3–N2	2.546(6)
Ca1–N1	2.820(6)	Ca3–N2	2.593(3)
		Ca3–N1	2.659(6)
		Ca3–N2	2.764(2)
		Ca3–N1	2.773(6)

Supplementary Table 5: Selected bond angles (in deg.) occurring in Ca_4FeN_4.

N1–Fe1–N1	117.8(4)				
N1–Fe1–N3	121.08(18)				
N1–Fe1–N3	121.09(18)				
N3–Ca1–N3	90.44(7)	N3–Ca2–N2	180	N1–Ca3–N2	168.85(16)
N3–Ca1–N3	179.39(13)	N3–Ca2–N1	94.81(12)	N1–Ca3–N2	87.57(17)
N3–Ca1–N3	90.13(3)	N2–Ca2–N1	85.19(12)	N2–Ca3–N2	92.27(9)
N3–Ca1–N3	90.13(3)	N3–Ca2–N1	94.81(12)	N1–Ca3–N1	92.47(18)
N3–Ca1–N3	179.39(13)	N2–Ca2–N1	85.19(12)	N2–Ca3–N1	98.58(15)
N3–Ca1–N3	89.30(7)	N1–Ca2–N1	170.4(2)	N2–Ca3–N1	83.08(16)
N3–Ca1–N1	70.23(17)	N3–Ca2–N1	94.75(11)	N1–Ca3–N2	83.16(16)
N3–Ca1–N1	90.09(17)	N2–Ca2–N1	85.25(11)	N2–Ca3–N2	88.65(9)
N3–Ca1–N1	109.56(17)	N1–Ca2–N1	89.75(9)	N2–Ca3–N2	131.58(17)
N3–Ca1–N1	90.30(17)	N1–Ca2–N1	89.46(9)	N1–Ca3–N2	144.52(16)
N3–Ca1–N1	90.09(17)	N3–Ca2–N1	94.75(11)	N1–Ca3–N1	90.09(17)
N3–Ca1–N1	70.23(17)	N2–Ca2–N1	85.25(11)	N2–Ca3–N1	95.70(14)
N3–Ca1–N1	90.30(17)	N1–Ca2–N1	89.46(9)	N2–Ca3–N1	148.99(16)
N3–Ca1–N1	109.56(17)	N1–Ca2–N1	89.75(9)	N1–Ca3–N1	66.1(2)
N1–Ca1–N1	152.3(2)	N1–Ca2–N1	170.5(2)	N2–Ca3–N1	78.64(15)

Supplementary Table 6. Distortions in the CaN6 octahedra. Displayed are quadratic elongation, bond angle variance and effective coordination number calculated with VESTA.[6,12]

Polyhedron	Quadratic Elongation	Bond angle variance / deg.	ECoN
**$Ca1N_6$**	1.0508	140.62	5.03
**$Ca2N_6$**	1.0064	16.77	5.74
**$Ca3N_6$**	1.0738	240.61	5.52

Supplementary Table 7. Crystallographic data for the Rietveld refinement of laboratory X-ray and neutron powder diffraction data.

	**X-ray RT**	**Neutron 50 K**	**Neutron 1.5 K**
**Crystal Data**			
Formula		Ca_4FeN_4	
Crystal system		Orthorhombic	
Space group		*Ibca* no. 73	
Cell parameters, Å	*a* = 6.939(1)	*a* = 6.950(1)	*a* = 6.947(1)
	b = 6.959(1)	*b* = 6.931(1)	*b* = 6.932(1)
	c = 22.538(1)	*c* = 22.526(1)	*c* = 22.528(1)
Cell volume, $Å^3$	1088.85(1)	1085.15(1)	1085.00(1)
Formula units		8	
**Data Collection**			
Diffractometer	Stoe StadiP	ISIS WISH Beamline	
Radiation	Mo-Kα1	Thermal neutron TOF	
Temperature, K	297(1)	50	1.5
Resolution, Å	20.3–0.6	12.1-1.1	12.1-1.4
**Refinement**			
Number of parameters	32	36	58
Constraints	0	2	2
Program	Topas Academic V4.1	GSAS-II	
Background function		Shiften Chebychev	
Rwp, %	5.5	2.9	2.8

Supplementary Table 8. Atomic displacements by comparison of the RT single-crystal model with the nuclear models at 50 and 1.5 K obtained with neutron powder diffraction. Atomic displacements u_x, u_y, u_z are given in relative units, $|u|$ in Å. d_{max} and d_{avg} (in Å) give the maximum displacement and average displacement of an atom pair. Data were calculated with the COMPSTRU tool of the Bilbao crystallographic server.[25]

	Atomic Displacements			
Atom	u_x	u_y	u_z	$\|u\|$ / Å
**50 K**				
Fe1	0.0000	0.0000	0.0022	0.0485
Ca1	0.0000	0.0055	0.0000	0.0378
Ca2	0.0000	0.0000	−0.0004	0.0095
Ca3	0.0135	−0.0321	−0.0014	0.02428
N1	−0.0348	−0.0095	0.0017	0.2522
N2	0.0000	0.0000	0.0067	0.1509
N3	0.0000	0.0000	0.0014	0.0318
d_{max}				0.2522
d_{avg}				0.1409
**1.5 K**				
Fe1	0.0000	0.0000	0.0034	0.0762
Ca1	0.0000	0.0083	0.0000	0.0574
Ca2	0.0000	0.0000	0.0002	0.0054
Ca3	0.0241	−0.0372	−0.0018	0.3091
N1	−0.0318	−0.0116	0.0037	0.2481
N2	0.0000	0.0000	0.0083	0.1872
N3	0.0000	0.0000	0.0024	0.0544
d_{max}				0.3091
d_{avg}		–		0.1661

Supplementary Table 9: Data obtained from EDX analysis. The experimentally obtained unit formula normed on Ca with standard deviation is shown as well as the deviation in percent.

**Datapoint**	**N / at-%**	**Ca / at-%**	**Fe / at-%**
1	38.92	48.14	12.94
2	36.17	49.93	13.90
3	33.43	51.41	15.16
4	43.66	43.76	12.58
5	39.36	47.60	13.03
6	39.70	47.31	12.98
7	50.32	38.96	10.72
8	51.88	38.00	10.12
9	40.28	46.31	13.41
10	54.44	35.35	10.21
11	49.39	39.75	10.86
12	49.55	39.22	11.23
13	54.20	36.09	9.71
14	40.60	47.39	12.02
15	48.92	41.52	9.56
**Mean / at-%**	44.72	43.38	11.90
**Standard deviation / at-%**	6.84	5.27	1.69
**Unit formula**	4.12	4.00	1.10
**Unit formula standard deviation**	0.58	0.44	0.14
**Deviation / %**	0.14	0.11	0.13

Supplementary Table 10: Bond valence sum calculations with bond valence parameters $R_{Fe\text{–}N}$ =1.86 Å and $R_{Ca\text{–}N}$ =2.14 Å (from Brese and O'Keeffe). Bond valences v_{ij} for each bond and bond valence sum V_i are calculated for the first coordination sphere of each cation.[26]

**Cation-anion pair**	**Bond Distance / Å**	v_{ij}	$V_i = \Sigma v_{ij}$
Fe1–N1	1.731	1.417	
Fe1–N3	1.758	1.317	
Fe1–N1	1.731	1.417	**4.15**
Ca1–N3	2.455	0.427	
Ca1–N1	2.821	0.159	
Ca1–N3	2.431	0.455	
Ca1–N1	2.821	0.159	
Ca1–N3	2.455	0.427	
Ca1–N3	2.431	0.455	**2.08**
Ca2–N1	2.619	0.274	
Ca2–N1	2.651	0.251	
Ca2–N1	2.651	0.251	
Ca2–N3	2.441	0.443	
Ca2–N2	2.527	0.351	
Ca2–N1	2.619	0.274	**1.85**
Ca3–N2	2.593	0.294	
Ca3–N1	2.474	0.405	
Ca3–N1	2.658	0.247	
Ca3–N2	2.546	0.334	
Ca3–N1	2.772	0.181	
Ca3–N2	2.764	0.185	**1.65**

5. Supplementary References

1. Huppertz, H. Multianvil high-pressure / high-temperature synthesis in solid state chemistry. *Z. Kristallogr.* **219**, 330–338 (2004).

2. Bruker-AXS, *APEX3 - Crystallography Software Suite*. (Karlsruhe, 2016).

3. Bruker-AXS, *XPREP Reciprocal Space Exploration*. (Karlsruhe, 2001).

4. Sheldrick, G. M. *SHELXS - A Program for Crystal Structure Solution*. (Göttingen, 1997).

5. Farrugia, L. J. WinGX and ORTEP for Windows : an update. *J. Appl. Cryst.* **45**, 849–854 (2012).

6. Momma, K. & Izumi, F. VESTA3 for three-dimensional visualization of crystal, volumetric and morphology data. *J. Appl. Cryst.* **44**, 1272–1276 (2011).

7. Coelho, A. A. *TOPAS-Academic*. (Brisbane, 2007).

8. Chapon, L. C. *et al.* Wish: The New Powder and Single Crystal Magnetic Diffractometer on the Second Target Station. *Neutron News* **22**, 22–25 (2011).

9. Toby, B. H. & Von Dreele, R. B. GSAS-II : the genesis of a modern open-source all purpose crystallography software package. *J. Appl. Cryst.* **46**, 544–549 (2013).

10. Farrugia, L. J. *PLATON*. (University of Glasgow, Glasgow, 1995).

11. Bruker-AXS, *SADABS*. (Karlsruhe, 2001).

12. Robinson, K., Gibbs, G. V. & Ribbe, P. H. Quadratic Elongation: A Quantitative Measure of Distortion in Coordination Polyhedra. *Science* **172**, 567–570 (1971).

13. Vennos, D. A. & DiSalvo, F. J. Synthesis and characterization of a new ternary nitride, Ca_3VN_3. *J. Solid State Chem.* **98**, 318–322 (1992).

14. Vennos, D. A., Badding, M. E. & DiSalvo, F. J. Synthesis, structure, and properties of a new ternary metal nitride, Ca_3CrN_3. *Inorg. Chem.* **29**, 4059–4062 (1990).

15. Tennstedt, A., Röhr, C. & Kniep, R. $Ca_3[MnN_3]$, ein neues Nitridomanganat(III): Anionen $[Mn^{III}N_3]^{6-}$ mit C_{2v}-Symmetrie. *Z. Naturforsch.* **48b**, 1831–1834 (1993).

16. Bendyna, J. K., Höhn, P. & Kniep, R. Crystal structure of tristrontium trinitridoferrate(III), $Sr_3[FeN_3]$. *Z. Kristallogr. - New Cryst. Struct.* **223**, 109–110 (2008).

17. Yee, K. A. & Hughbanks, T. Spin State Stabilities and Distortions of the Novel MN_3^{6-} (M = V, Cr, Fe) Ions. *Inorg. Chem.* **31**, 1921–1925 (1992).

18. Alvarez, S. Bonding and stereochemistry of three-coordinated transition metal compounds. *Coord. Chem. Rev.* **193–195**, 13–41 (1999).

19. Jansen, N. *et al.* Mössbauer Spectroscopy and Electronic Structure Calculations of the Nitridoferrates(III): $Li_3[FeN_2]$ and $Ba_3[FeN_3]$. *Angew. Chem. Int. Ed. Engl.* **31**, 1624–1626 (1992).

20. Höhn, P., Kniep, R. & Rabenau, A. $Ba_3[FeN_3]$: Ein neues Nitridoferrat(III) mit $[CO_3]^{2-}$-isosteren Anionen $[FeN_3]^{6-}$. *Z. Kristallogr.* **196**, 153–158 (1991).

21. Barker, M. G., Begley, M. J., Edwards, P. P., Gregory, D. H. & Smith, S. E. Synthesis and crystal structures of the new ternary nitrides Sr_3CrN_3 and Ba_3CrN_3. *J. Chem. Soc. Dalton Trans.* 1–5 (1996).

22. Hursthouse, M. B. & Rodesiler, P. F. Crystal and molecular structure of tris(hexamethyldisilylamido)iron(III). *J. Chem. Soc. Dalton Trans.* 2100–2012 (1972).

23. Hendricks, S. B. & Kosting, P. R. The Crystal Structure of Fe_2P, Fe_2N, Fe_3N and FeB. *Z. Kristallogr., Kristallgeom., Kristallphys., Kristallchem.* **74**, 511–533 (1930).

24. Ovchinnikov, A. *et al.* $Ca_{12}[Mn_{19}N_{23}]$ and $Ca_{133}[Mn_{216}N_{260}]$: Structural Complexity by 2D Intergrowth. *Angew. Chem. Int. Ed.* **57**, 11579–11583 (2018).

25. de la Flor, G., Orobengoa, D., Tasci, E., Perez-Mato, J. M. & Aroyo, M. I. Comparison of structures applying the tools available at the Bilbao Crystallographic Server. *J. Appl. Cryst.* **49**, 653–664 (2016).

26. Brese, N. E & O'Keeffe, M. Bond-Valence Parameters for Solids. *Acta Crystallogr., Sect. B: Struct. Sci.* **47**, 192-197 (1991).
